# Molecular mechanisms involved in podocyte EMT and concomitant diabetic kidney diseases: an update

**DOI:** 10.1080/0886022X.2017.1313164

**Published:** 2017-04-17

**Authors:** Qidi Ying, Guanzhong Wu

**Affiliations:** Department of Pharmacology, Pharmacy, China Pharmaceutical University, Nanjing, Jiangsu, China

**Keywords:** Podocyte, epithelial–mesenchymal transition, molecular mechanism, diabetic kidney disease, hyperglycemia

## Abstract

Epithelial–mesenchymal transition (EMT) is a tightly regulated process by which epithelial cells lose their hallmark epithelial characteristics and gain the features of mesenchymal cells. For podocytes, expression of nephrin, podocin, P-cadherin, and ZO-1 is downregulated, the slit diaphragm (SD) will be altered, and the actin cytoskeleton will be rearranged. Diabetes, especially hyperglycemia, has been demonstrated to incite podocyte EMT through several molecular mechanisms such as TGF-β/Smad classic pathway, Wnt/β-catenin signaling pathway, Integrins/integrin-linked kinase (ILK) signaling pathway, MAPKs signaling pathway, Jagged/Notch signaling pathway, and NF-κB signaling pathway. As one of the most fundamental prerequisites to develop ground-breaking therapeutic options to prevent the development and progression of diabetic kidney disease (DKD), a comprehensive understanding of the molecular mechanisms involved in the pathogenesis of podocyte EMT is compulsory. Therefore, the purpose of this paper is to update the research progress of these underlying signaling pathways and expound the podocyte EMT-related DKDs.

## Introduction

As a severe kidney complication of diabetes mellitus (DM), diabetic kidney disease (DKD) is the major cause of disability and death in patients with diabetes. Intuitively, renal fibrosis and proteinuria are two of the most significant pathological changes of DKD. Recent findings suggest that numerous pathways are activated during the course of DKD and that these pathways individually or collectively play a role in the induction and progression of renal failure.^1^ The podocyte is a highly-differentiated kidney cell situated on the outer surface of glomerular basement membrane (GBM), which forms a vital component of the glomerular filtration barrier (GFB). Podocyte is the fundamental constituent of GFB and plays an important role in the existence of its specific molecular and charge characteristics.^2^ Hyperglycemia has been found to cause morphological changes of podocyte, most of which could be described as an epithelial–mesenchymal transition (EMT). Hyperglycemia has been demonstrated to incite podocyte EMT through several molecular mechanisms such as TGF-β/Smad classic pathway, Wnt/β-catenin signaling pathway, Integrins/ILK signaling pathway, RTK/Ras/Erk signaling pathway, MAPKs signaling pathway, PI3K/AKT/mTOR signaling pathway, Jagged/Notch signaling pathway, and NF-κB signaling pathway.^3–7^ Recent studies have confirmed that the Smad classic pathway and multiple other pathways are jointly involved in the inducement of podocyte EMT process. Moreover, crosstalk and interaction between these pathways make podocyte EMT molecular mechanism even more complex and controversial.^8^ The purpose of this paper is to update the research progress of these underlying signaling pathways and expound podocyte EMT-associated DKD.

## The specialization of podocyte

**Figure 1.**
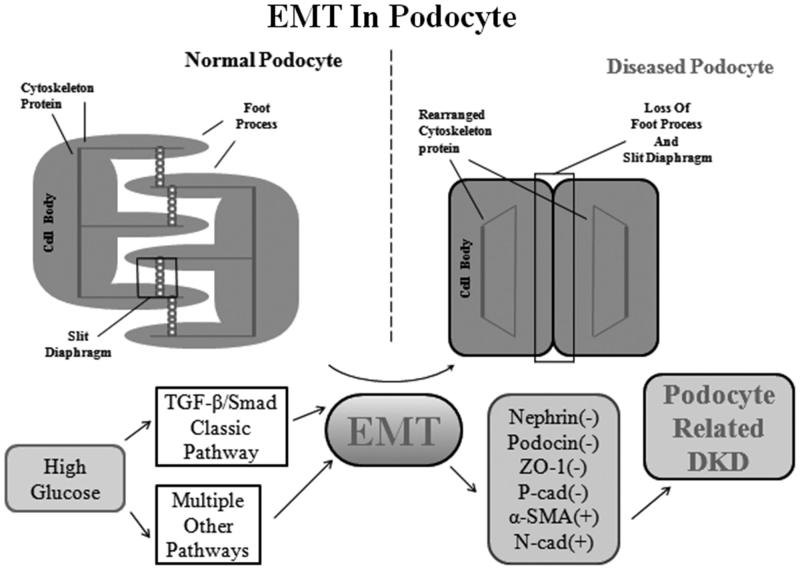
EMT process in podocyte. The podocyte comprises three main compartments, the cell body, the major processes, and the foot processes. Each of these segments shares a common actin cytoskeleton. Neighboring foot processes are regularly interdigitated and bridged by a specialized cell–cell junction known as the slit diaphragm. Podocytes can be injured by hyperglycemia through TGF-β/Smad classic pathway and multiple other pathways. Following EMT process, the podocyte foot processes are effaced, which results in a loss of the slit diaphragm. The expression of nephrin, podocin, P-cadherin and ZO-1 is downregulated, the actin cytoskeleton is rearranged and the podocyte is no longer able to restrict urinary protein loss. This EMT process can finally cause podocyte-related diabetic kidney diseases (DKD).

The podocyte is a kind of terminally differentiated cells which covers the GBM over the glomerular capillary. It consists of three morphologically and functionally different segments, the cell body, the major processes and the extending fingerlike foot processes (FPs).^9^ Podocytes sustain the glomerular structural integrity by counteracting the outward expansion of the GBM, for which the FPs is the main functional unit of the podocyte. These contain loops of filamentous actin (F-actin) that can be assembled, disassembled, and bundled together in response to the changing requirements of the foot process. The tensile strength of F-actin and its concentration in the FPs enable the podocyte to withstand the pressure of glomerular flow.^10^ The FPs of neighboring podocytes are connected by a continuous adherent junction structure named the slit diaphragm (SD), which forms the huge filtration surface of GFB. The extracellular SD is linked to the intracellular one, a highly dynamic cytoskeleton through adaptor proteins. These adaptor proteins, such as CD2-associated protein (CD2AP), zonula occludens 1 (ZO-1) and β-catenin, located at the intracellular SD insertion area near lipid rafts, have important structural and functional roles.^11^^,^^12^ Recently, it has been widely accepted that a huge loss of the highly specialized podocyte structure limits the capacity of the final layer of the GFB to restrict urinary protein loss. These phenomena observed in diseased podocytes could be due to a result of EMT (Figure 1).

Epithelial–mesenchymal transition is a tightly regulated process by which epithelial cells lose their hallmark epithelial characteristics and gain the features of mesenchymal cells. For podocytes, expression of nephrin, podocin, P-cadherin and ZO-1 is downregulated, the cell-to-cell junctions (SD) will be altered, and the actin cytoskeleton will be rearranged.^13^ The SD is a consummate adherens junction served as a size-selective barrier and is linked to the actin-based cytoskeleton by adaptor proteins, including CD2AP, ZO-1, and β-catenin.^11^^,^^14^ As important SD proteins, the loss of nephrin, podocin and P-cadherin expression is detrimental to the phenotype and function of the podocyte.

Epithelial–mesenchymal transition in podocytes indicates a serious of differentiation compared with general epithelial cells mostly whereby the specificity of SD adaptor proteins. For instance, one of the criteria for type-2 EMT is a cadherin switch from E-cadherin to N-cadherin. While the podocytes do undergo a cadherin switch in EMT diseased state, this is from P-cadherin to N-cadherin. Other characteristic signals, such as high cell matrix interactions and high migration capacity, also show remarkable difference against typical epithelial cells. Because of these specific points, some scientists claim that the concept of EMT as an important pathomechanism driving podocyte injury is highly controversial. From their idea, differentiated podocytes are visceral epithelial cells that arise from epithelial precursors during renal development. Mature podocytes lack classic characteristics of epithelial cells (e.g., they have lost their mitotic activity and tight junctions, and cell bodies are independent of each other) and they have established features of mesenchymal cells (such as expression of vimentin). Therefore, podocyte differentiation involves a physiological EMT, and it is unclear how differentiated podocytes could undergo EMT. For example, May et al.^15^ demonstrated in his review that the podocytes do not undergo a typical type-2 EMT; so instead, the new term “podocyte disease transformation” has been coined in order to distinguish this process from EMT.

However, recently most of the scientists prefer to regard podocyte dedifferentiation as a process of EMT. Actually, differentiated podocyte still indicates most of the epithelial cell characters. Differentiated podocyte is polarized with low invasive capacity and anchorage dependence. Its SD is a functional-modified tight junction. Additionally, podocyte do undergo an analogous type-2 EMT in response to hyperglycemia and TGF-β. Li^16^, Yamaguchi^17^, and Kretzler^18^ study all independently indicated a loss in nephrin and ZO-1 expression in glomerular podocytes, whereas key EMT regulators, such as Snail and ILK expression were significantly increased in DKD patients, suggesting an active EMT formation in podocytes. Ghiggeri^19^ findings corroborated the hypothesis of podocyte EMT as a putative mechanism underlying the sclerotic reactions and genetic ablation of nephrin induced a constitutional EMT as well. A recent study in 2016 proved the function of GSK-3β both in podocyte dysfunction and EMT, which emphasized the hallmarks podocytes lost when undergoing EMT.^20^

In conclusion, it is widely accepted recently that podocytes do undergo EMT process under high glucose condition. Actually, the specialization of podocyte EMT also increases the research value of podocytes.^15^

## Molecular mechanisms in podocyte EMT by hyperglycemia

**Figure 2.**
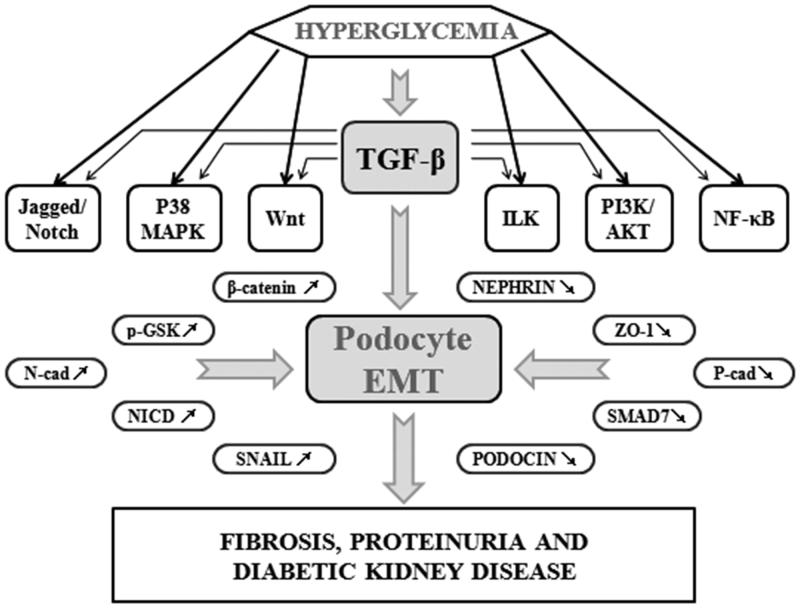
Molecular mechanisms involved in induction and progression of podocyte EMT caused by hyperglycemia. As can be seen in this figure, high glucose could cause podocyte EMT through multiple signaling pathways like TGF-β/Smad pathway, Wnt pathway, ILK pathway, p38/MAPK pathway, PI3K/AKT pathway, Jagged/Notch pathway and NF-κB pathway, among which TGF-β is the essential cytokine which plays a vital role. The expression of many signaling molecules are disordered, like the downregulation of nephrin, podocin, P-cad, Smad7, ZO-1, and the upregulation of β-catenin, Snail, p-GSK, NICD, N-cad, which leads to podocyte EMT and results in fibrosis, proteinuria and kidney diseases.

High glucose, *in vitro* and *in vivo*, could be the inducible factor of podocyte EMT through TGF-β/Smad classic pathway and multiple other pathways such as Wnt/β-catenin signaling pathway, Integrins/ILK signaling pathway, MAPKs signaling pathway, PI3K/AKT/mTOR signaling pathway, Jagged/Notch signaling pathway, and NF-κB signaling pathway (Figure 2). Moreover, other detrimental factors, such as hyperlipidemia, advanced oxidative products and advanced glycation end products, have currently been proved to play a similar role. Additionally, endocrine disorder caused by high glucose level, such as the upregulation of renin, aldosterone and *Ang*II, is also the fundamental factor which induces podocyte dedifferentiation and DKD.^21^ This review expounds the pivotal pathways in podocyte EMT in order to help give raise to potential therapeutic strategies to inhibit DKD and the following end-stage renal diseases (ESRD).

### TGF-β/Smad classic pathway

Upregulation of transforming growth factor-β (TGF-β) in podocytes is observed in patients with most progressive kidney diseases, indicating that TGF-β is the key regulator of ECM protein synthesis in podocytes. During EMT process, TGF-β1 contributes to excessive deposition of fibrotic materials, rewriting the expression of several epithelial cell recognition and organizational proteins.^22^ Smad-dependent pathways are predominantly mediated pathways for TGF-β1. Smads are subdivided into three classes: receptor-regulated Smads (R-Smads, Smad1, 2, 3, 5, and 8), common Smads (Co-Smad and Smad4) and inhibitory Smads (I-Smad, Smad6, and 7).^23^ Following TβRII activation, R-Smads form oligomeric complexes with the common Smad prior to translocation into the nucleus and regulation of gene transcription.^24^

**Figure 3.**
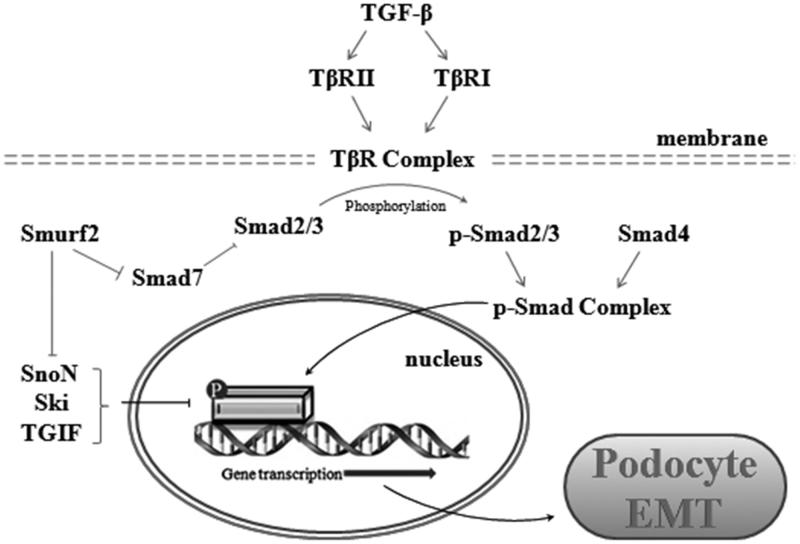
TGF-β/Smad classic pathway. Activated TGF-β first integrates TGF-β receptor type II (TβRII) and TGF-β receptor type I (TβRI) to form ligand receptor complex. This association results in the downstream phosphorylation and activation of Smad2 and Smad3. Phosphorylated Smad2/3 combines with Smad4 to form a Smad complex in cytoplasmic domain and gets nuclear translocation. After translocated into nucleus, the adjusted Smad complex is integrated with EMT-related Smad binding element in gene promoter region to play regulatory roles. Simultaneously, activated TGF-β can also upregulates Smad ubiquitination regulatory factor-2 (Smurf2), an ubiquitin ligase which can degrade TGF-β pathway inhibitors like Smad7, SnoN, Ski, and TGIF. Through this two aspects, activated TGF-β can lead to podocyte EMT independently.

The classic signaling pathway of TGF-β in EMT is as follows. TGF-β first integrates TGF-β receptor type II (TβRII) and TGF-β receptor type I (TβRI) to form ligand receptor complex. Binding of TGF-β1 to the TβRII receptor is accompanied by phosphorylation and subsequent activation of TβRI within its cytoplasmic domain. This association results in the downstream phosphorylation and activation of its classic signaling mediators, Smad2 and Smad3. Phosphorylated Smad2/3 combines with Smad4 to form a Smad complex in cytoplasmic domain and get nuclear translocation.^13^ After translocated into nucleus, the adjusted Smad complex is integrated with EMT-related Smad binding element in gene promoter region to play regulatory roles. The expression of EMT-related genes, like α-smooth muscle actin (α-SMA), connective tissue growth factor (CTGF), matrix metalloproteinases-9 (MMP-9), Snail, Smad interacting protein 1 (SIP1) and hairy/enhancer-of-split-related with YRPW motif 1 (HEY1) are all upregulated by Smad complex.^6^ In podocyte, a considerable part of these target genes are the inhibitors of P-cadherin (P-cad). These genes induce cell phenotype changes by inhibiting the expression of P-cad. The rest are signal molecules which contact different signaling pathways to play fundamental regulatory roles during podocyte EMT process (Figure 3).

In TGF-β/Smad classic pathway, the I-Smads inhibit R-Smads phosphorylation by blocking their access to TβRI and by promoting the degradation of the receptor complexes. The co-repressors SnoN (Ski-related novel gene and non-*Alu*-containing), Ski (Sloan-Kettering Institute proto-oncogene), and TGIF (TG-interacting factor) prevent gene transcription through inhibition of R-Smads.^25^^,^^26^ However, Smad ubiquitination regulatory factor-2 (Smurf2) is an ubiquitin ligase that specifically targets certain members of Smad proteins for degradation such as Ski, SnoN, and TGIF.^27^^,^^28^ Overexpression of Smurf2 and enhanced co-repressor degradation^9^ is also a part of TGF-β induced podocyte EMT process.

Without the classic pathway, TGF-β could also act as a predominant factor to most of the podocyte-related DKD signaling pathways. As what had been shown in Figure 2, TGF-β could individually trigger all the key events of podocyte EMT completely with or without the existence of hyperglycemia. Recent study clearly indicated that TGF-β could stimulate the expression of CTGF to induce sustained fibrosis and to exacerbate ECM production.^29^ Additionally, Ras/Raf/Erk pathway could also be activated by autophosphorylation of TβRII. After the stimulation of TGF-β1, TβRII, and TβRI were phosphorylated successively to form a heterologous tetramer, which motivated the expression of associated cytokines like Src homology cytokine (Shc), growth factor receptor binding protein 2 (Grb2), and son of sevenless (Sos) to promote the formation of ShcA/Grb2/Sos complex. ShcA/Grb2/Sos complex then caused sequential activation of Ras/Raf/MEK/Erk, p-Erk translocated to the nucleus and activated target gene to cause podocyte EMT.^30^

### Wnt/β-catenin signaling pathway

Wnt signaling pathway is an essential signaling pathway in living organisms. Glycosylated cysteine-rich Wnt proteins play a variety of cellular functions involving cell fate, proliferation, migration, polarity, and death^31–33^ through at least three distinct intracellular pathways, including the canonical Wnt/β-catenin signaling pathway, the non-canonical Wnt/Ca^2+^ pathway, and Wnt/planar cell polarity (PCP) pathway^34^. Canonical Wnt/β-catenin signaling pathway expression is increased in podocytes of hyperglycemic patients as well as mouse models and plays a critical role in integrating cell differentiation EMT.^35^

In canonical Wnt/β-catenin signaling pathway, β-catenin protein is inhibited by the Axin complex in the cytoplasm without the Wnt signaling factor. Axin complex is consisted of the axis protein Axin, the tumor gene suppressor adenomatosis polyposis coli (APC), casein protein kinase 1 (CK1) and glycogen synthase kinase-3β (GSK-3β), among which CK1 and GSK-3β may cause β-catenin amino-terminal phosphorylation and inactivation. When phosphorylated, β-catenin can be recognized and led to ubiquitin-mediated proteasomal degradation by β-TrCP.^36^ The continuous decline of β-catenin makes it impossible to get nuclear translocation and reach the target gene to play a role. Under this circumstance, the target gene of Wnt/β-catenin signaling pathway is inhibited of T cell factor/lymphoid enhancer factor (TCF/LEF) protein family and is unable to express.^37^^,^^38^ In contrast, when Wnt signaling is activated by high glucose condition or various intercellular stimulators like TGF-β, Wnt ligands can combine with transmembrane protein Frizzled (FZD) receptors and low density lipoprotein receptor-related protein (LRP6) or its analog (LRP5) to activate Wnt signaling pathway. Wnt–FZD–LRP5/6 complex is then phosphorylated and activated by Disheveled (Dvl). Activated Wnt–FZD–LRP5/6 complex targets Axin complex, leading to the phosphorylation of GSK-3β and dephosphorylation of β-catenin, which causes the accumulation of β-catenin in podocyte cytoplasm. Subsequently, accumulated β-catenin is translocated to the nucleus and activates Wnt target gene with TCF/LEF. The expression products of Wnt target gene participate in a variety of physiological mechanisms *in vivo* to cause podocyte EMT.^39^^,^^40^

Activation of Wnt signaling and over expression of β-catenin has been reported in podocyte EMT-related experiments *in vitro* and *in vivo*, most of which is interacted with other signal pathways. ILK dependency β-catenin nuclear translocation was observed in both puromycin (PAN) intervened podocyte and FSGS samples.^41^^,^^42^ TGF-β and doxorubicin might also adjust Wnt/β-catenin signaling pathway *in vitro*. Mice were injected with doxorubicin and Wnt1 plasmid intravenously to construct classic podocyte EMT injury models and β-catenin activation animal models. Downregulation of glomerular SD proteins (nephrin) and proteinuria was observed in model mice.^43^*In vitro* model, intervening podocytes with recombinant Wnt3a could cause activation of β-catenin accompanying the decline in adhesion between podocytes and collagen I/IV, which demonstrated the existence of podocyte EMT.^35^ In podocyte-specific β-catenin overexpression transgenic mice, podocyte EMT resulted in phenotypic changes and decreased adhesion to GBM by integrin β1, leading to podocyte off, proteinuria, and DKD. Conditional knockout of β-catenin in podocyte had protective effect of doxorubicin damage by the improvement of nephrin and podocin level and suppression of proteinuria and fibrosis, which was similar with the injection of Wnt pathway inhibitor Dickkopf-related protein 1 (Dkk1).^44^ These current results suggested that intervention to β-catenin might have potential clinical value in podocyte EMT.

### Integrins/ILK signaling pathway

**Figure 4.**
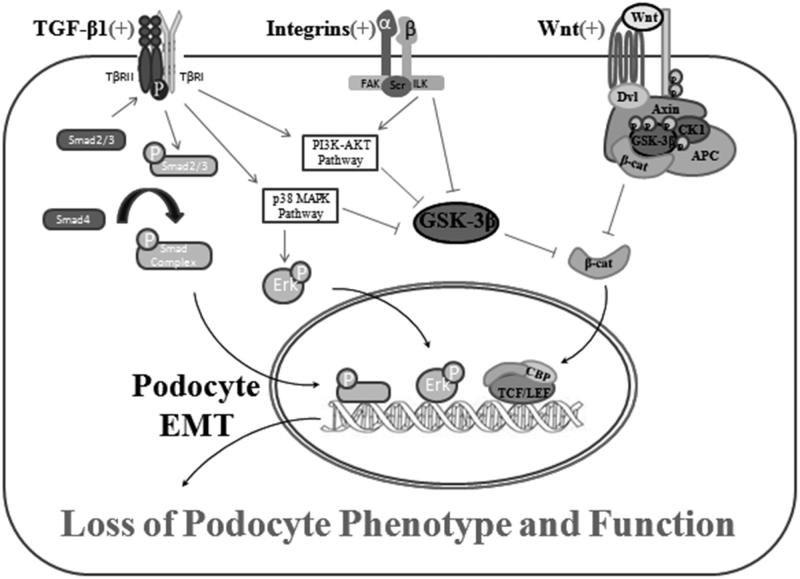
Cross-link between TGF-β pathway, Wnt pathway, and ILK pathway. This simplified schematic shows major intracellular signaling networks and mediators involved in the regulation of podocyte EMT. Overall, TGF-β is the prototypic inducer and TGF-β/Smad pathway is the essential one of podocyte EMT, whereas the effects of other mediators are often context-dependent, variable, and incomplete. Activated ILK directly phosphorylates several physiologically important downstream effector kinases including Akt and GSK-3β, leading to the stabilization of β-catenin, which indirectly controls the expression of an array of genes that are required for the EMT process. As to Wnt network, upon binding to Wnt receptor, activated Wnt proteins induce a series of downstream signaling events involving Disheveled (Dvl), axin, adenomatosis polyposis coli (APC), casein kinase-1 (CK-1), and GSK-3β, resulting in dephosphorylation of β-catenin. TGF-β, ILK, and Wnt signals are interconnected and converged at the phosphorylation of GSK-3β and activation of β-catenin, which leads to the activation of EMT transcriptional programs. Therefore, β-catenin, to some extent, could function as a master switch that can integrate signal inputs from multiple pathways and control the EMT-related transcriptome.

Integrin-linked kinase is an intracellular serine/threonine protein kinase which interacts with the cytoplasmic domains of the β-integrins and cytoskeleton to mediate the integrin, growing factor, and Wnt signaling pathways in podocyte transdifferentiation.^45^ The biologic activity of ILK has recently been concluded into two principal properties: a scaffolding protein and a protein kinase.^46^^,^^47^ As a scaffolding protein, ILK interacts with integrins, particularly interesting new cysteine-histidine rich protein (PINCH) and SD to build a molecular bridge which connects the cell–matrix integrin signaling with SD signaling.^48^ Conditional knockout of ILK in a podocyte-specific manner results in massive proteinuria, fibrosis, glomerulosclerosis, and premature death in mice.^49^^,^^50^ As a protein kinase, ILK directly phosphorylates several physiologically essential downstream effector kinases like Akt, p38 MAPK, and GSK-3β, resulting to the stabilization and accumulation of β-catenin, which controls the expression of a series of target genes that are irreplaceable for podocyte EMT progress (Figure 4).

The effect of ILK has recently been identified through the interactions with other classic signaling pathways such as TGF-β/Smad and Wnt/β-catenin signaling pathway. Under high glucose condition, ILK could individually or cooperatively highlight the expression of α-SMA in podocyte, which is a remarkable index of mesenchymal cells. Upregulated level of ILK and α-SMA are both significant hallmarks in the progression of podocyte EMT.^51^ Li’s research illustrated that the expression of ILK is partly regulated by TGF-β/Smad signaling pathway, over-expression of ILK *in vivo* triggered the integral incident of EMT process. Indubitably, inhibition of ILK expression could also obstruct podocyte EMT and the coming DKD induced by TGF-β.^52^ LEE’s research also proved ILK as a vital factor for TGF-β induced EMT evolution.^53^ Recently, Laura AM’s research on both diabetic rats and diabetic pigs determined that blocking of αVβ3 integrin ligand occupancy could inhibit the progression of albuminuria and the development of fibrosis, which on the other hand symbolized the significance of integrins/ILK pathway during the process of podocyte-associated DKD.^54^^,^^55^

### MAPKs signaling pathway

Mitogen-activated protein kinases (MAPKs) are the common pathway for the whole physiological reactions from extracellular stimuli to nuclear transcription. By influencing the gene transcription and regulation, MAPKs thereby affect the biological behavior of cells, including inflammation, cell differentiation, cell growth, and cell death.^56^^,^^57^ C-Jun N-terminal kinase (JNK), also known as stress-activated protein kinase (SAPK), is a member of MAPK family. It have been found that JNK could be phosphorylated on Thr and Tyr residues to form three JNK isoforms, JNK1, JNK2, and JNK3 (also known as SAPKα, SAPKβ, and SAPKγ, respectively).^58^ The JNK isoforms are strongly activated in response to various cellular stresses, including oxidative stress, DNA-damaging agents, cytokines, UV irradiation, DNA and protein synthesis inhibitors, and growth factor deprivation.^59^ JNK is involved in cellular processes like cell proliferation and differentiation, cell apoptosis, cell movement, stress response and DNA damage repair, and has been reported to play an evident role in DKD.^60^ P38 MAPK is also a member of the MAPKs and is essential for the regulation of many cellular processes. It is a highly conserved signal transduction module monitoring cell proliferation and differentiation, cell migration, and inflammation^61^ by phosphorylate other cytoplasmic proteins. As a cytokine itself, p38 MAPK can translocate from the cytoplasm into the nucleus to regulate the activities of transcription factors.

Both JNK pathway and p38 MAPK pathway play a critical role in TGF-β induced podocyte EMT. Stambe et al. have shown that p38 MAPK could be a downstream signaling in TGF-β1 induced podocyte-related renal interstitial fibrosis (RIF). Blockade of p38 MAPK has been reported to reduce ECM production.^62^ Peinado et al.^63^ have shown that TGF-β1 could induce tubular EMT through the MAPK signal transduction pathway in dog renal tubular epithelial cells. Recent *in vitro* studies have shown that high levels of glucose could activate the p38 MAPK signaling pathway in podocytes and induce the phosphorylation of p38 MAPK, which promotes the production of fibronectin by the mesangial cells.^64^^,^^65^ JNK pathway is critical for the progression and maintenance of phenotypic and cellular changes-associated with EMT. A large fraction of invasive cells exhibited high JNK activity and low Smad signaling, whereas no such pattern was observed in noninvasive cells.^66^ Previous studies had also suggested that JNK activation may contribute to the TGF-β-induced gene regulation including expression of fibronectin and of other pro-fibrotic genes in podocytes.^67^ Actually, JNK signaling was observed to have no functional contribution in the onset of EMT process, but as cells progress through the mesenchymal state, JNK signaling gradually became a crucial transcriptional regulator of many genes which include activation of established mesenchymal genes and repression of known epithelial genes. As to podocyte EMT-related DKD, Wilmer et al.^65^ have found that JNK signaling activated could led to diabetic nephropathy and renal fibrosis. Kanta Taniguchi et al.^68^ also reported that chronic exposure to high glucose lead to abnormal increase of Src, which could cause podocyte EMT through p38 MAPK pathway. In conclusion, pathological level of JNK/p38 MAPK signaling is an EMT marker, upregulation of MAPKs signaling pathway is undoubtedly a predominant factor to podocyte EMT and its associated DKD.

### Jagged/Notch signaling pathway

**Figure 5.**
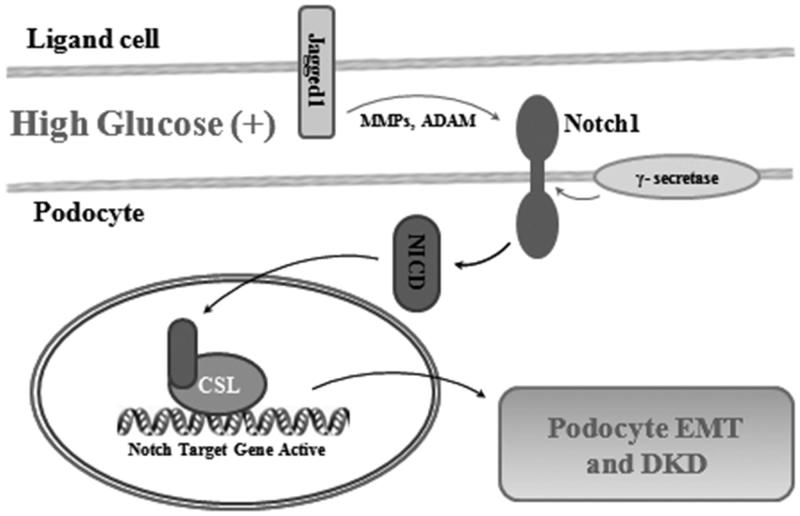
Jagged/Notch signaling pathway. Under high glucose condition, Notch receptor is firstly targeted with ligands binding to an adjacent receptor. Then the intramembrane Notch receptor (NICD) is cleaved by metalloproteinases and γ-secretase. The released NICD translocates to the nucleus and interacts with CSL to form a heteromeric complex which acts as an activator of target genes for podocyte EMT and DKD.

The Jagged/Notch signaling pathway is also considered an important regulator for EMT induction, despite several reports that Notch signaling is insufficient to completely induce EMT for it requires crosstalk with other signaling molecules.^69^ The Notch pathway is initiated through interactions between the Notch receptor and ligands binding to an adjacent receptor. Subsequently, the intramembrane Notch receptor (NICD) is cleaved by metalloproteinases and γ-secretase.^70^ The released NICD then translocates to the nucleus and interacts with C-protein binding factor 1/Suppressor of Hairless/Lag-1 (CBF1/Su(H)/Lag-1)^71^ to form a heteromeric complex and acts as an activator of target genes,^72^ including Hes and Hey (Figure 5). The Notch pathway maintains a balance between cell proliferation, differentiation, apoptosis, and plays an important role in determining cell fate and maintaining progenitor cell population. For example, in patients with chronic renal insufficiency, DKD, HIV nephropathy, FSGS, and systemic lupus erythematosus, Notch signaling pathway can be observed dramatically increased.^73–75^

Notch signaling requires coordination with other signals to promote podocyte EMT. Previous theories thought Notch signaling was a downstream target molecule of Wnt signaling pathway, Notch signaling pathway is a Wnt-mediated pathway, but recent studies have found that Notch is more like an independent podocyte EMT signaling pathway.^76^ Wnt and Notch pathways have also been indicated to cross-link between each other in order to induce a tumorigenic phenotype.^77^ TGF-β increases Notch activity through Smad 3, subsequently promoting Slug expression which suppresses P-cadherin.^78^ Slug-induced EMT is accompanied by the activation of β-catenin with Wnt pathway. Podocyte-specific Notch activation can cause dedifferentiation and shedding. In mouse models, pathological changes like proteinuria and rapidly progress FSGS could been observed following podocyte EMT, eventually cause kidney failure, and death.^79^^,^^80^ In different models, giving γ-secretase inhibitors or reducing Notch transcriptional binding protein levels can both significantly ameliorate glomerular injury and fibrosis,^81^^,^^82^ which on the other hand demonstrates the role of Notch in podocyte EMT.

### NF-κB signaling pathway

**Figure 6.**
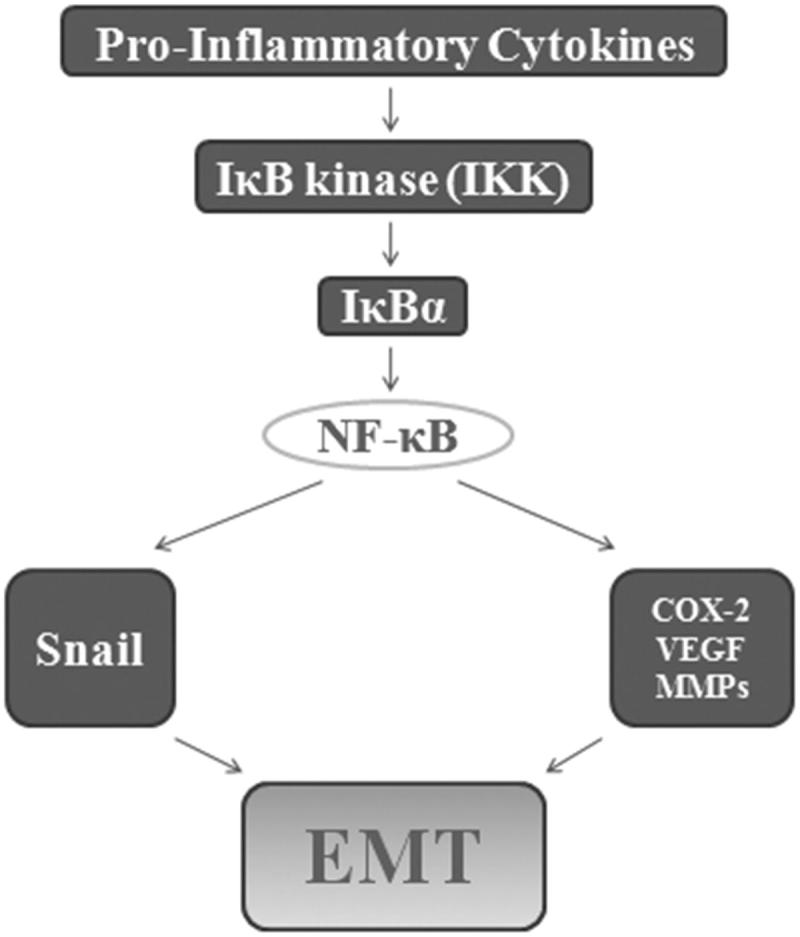
NF-κB signaling pathway. Stimulated by pro-inflammatory cytokines, IκB kinase (IKK) triggers the phosphorylation and degradation of IκBα. Liberated NF-κB then translocates to the nucleus to activate gene expression by recruiting transcriptional coactivators such.

As a transcription factor, NF-κB plays a key role in the expression of genes involved in EMT initiation and progression. NF-κB induces and maintains EMT in model systems through two mechanisms, upregulation of EMT master-switch transcription factors^28^^,^^83^^,^^84^ and stabilization of Snail.^85^ The NF-κB pathway is activated through IκB kinase (IKK). In unstimulated cells, inhibitory IκB subunits-associate with NF-κB and sequester them in the cytoplasm. Upon cellular stimulation by pro-inflammatory cytokines, IKK then triggers the phosphorylation and degradation of IκBα by the 26 S proteasome.^86^ Liberated NF-κB then translocates to the nucleus to activate gene expression by recruiting transcriptional coactivators.^87^ NF-κB promotes metastasis through the upregulation of several metastatic genes such as COX-2, metalloproteinases, VEGF, and most importantly the metastasis inducer Snail.^88^^,^^89^ Previous studies have shown that posttranslational modifications on Rel family member RelA are required for full NF-κB transcriptional activity^90^ (Figure 6).

NF-κB signaling pathway plays an important role in podocyte dedifferentiation and podocyte EMT. Qi et al.^91^ have found that NF-κB activation could significantly stimulate podocyte EMT, downregulate the expression of nephrin and podocin, subsequently leading to fibrosis and albuminuria in diabetic rats. Chung et al.^92^ also get close results in db/db mice that NF-κB could cause albuminuria through TNF-α/MCP-1 pathway.^92^ Podocyte-specific EMT alterations were again reproducible by cell junction rupture, induced by Ca^2+^ deprivation.^19^

## Conclusions

In this review, we discussed and summarized most of the known molecular mechanisms and downstream interactions between TGF-β/Smad classic pathway and multiple other pathways during the occurrence and development of podocyte EMT and associated DKD under high glucose condition. We also highlighted the significant cytokines linking these pathways to the process of EMT. As to podocytes, podocyte do undergo an analogous type-2 EMT in response to hyperglycemia and TGF-β. In other words, these special EMT also increase the research value of podocytes. Therefore, this review may a beneficial supplement for our further research on podocyte dysfunction and podocyte-related DKDs.
